# From skin testing to molecular diagnostics: the precision leap in dust mite allergy diagnosis and clinical translation challenges

**DOI:** 10.3389/falgy.2025.1598575

**Published:** 2025-06-05

**Authors:** Ming Han, Jindan Luo, Wenjing Zhou, Shuhui Wen, Yi Zhou, Yanjuan Ye, Xiaoli Ge

**Affiliations:** ^1^ Department of Pediatrics, The Affiliated Wuxi Second People’s Hospital of Jiangnan University, Wuxi Medical School, Jiangnan University, Wuxi, Jiangsu, China; ^2^ Department of Pediatrics, Wuxi Second People’s Hospital, Nanjing Medical University, Wuxi, Jiangsu, China; ^3^ Department of Pediatrics, The Affiliated Wuxi Second People’s Hospital of Jiangnan University, Wuxi, Jiangsu, China

**Keywords:** dust mite allergy, skin testing, molecular diagnostics, *in vivo* experiment, *in vitro* experiments, detection methods

## Abstract

Dust mites are ubiquitous in human living environments and represent the primary source of indoor air allergens worldwide. They are capable of triggering allergic rhinitis, conjunctivitis, asthma, atopic dermatitis, and other allergic conditions. Long-term avoidance of dust mite allergens should decrease sensitization, significantly improves skin lesions, and reduces both the development and severity of respiratory diseases. Therefore, early diagnosis of dust mite allergy is critical for effective treatment and intervention. This review summarizes the existing methods for detecting dust mite allergy, which include both *in vivo* and *in vitro* approaches—such as skin prick testing(SPT), atopy patch testing(APT), provocation tests, basophil activation test (BAT), and molecular component-resolved diagnostics(CRD)—and analyzes the underlying principles, advantages, and limitations of each method to serve as a reference for the development of future detection methods.

## 1 Introduction

**Figure 1:**
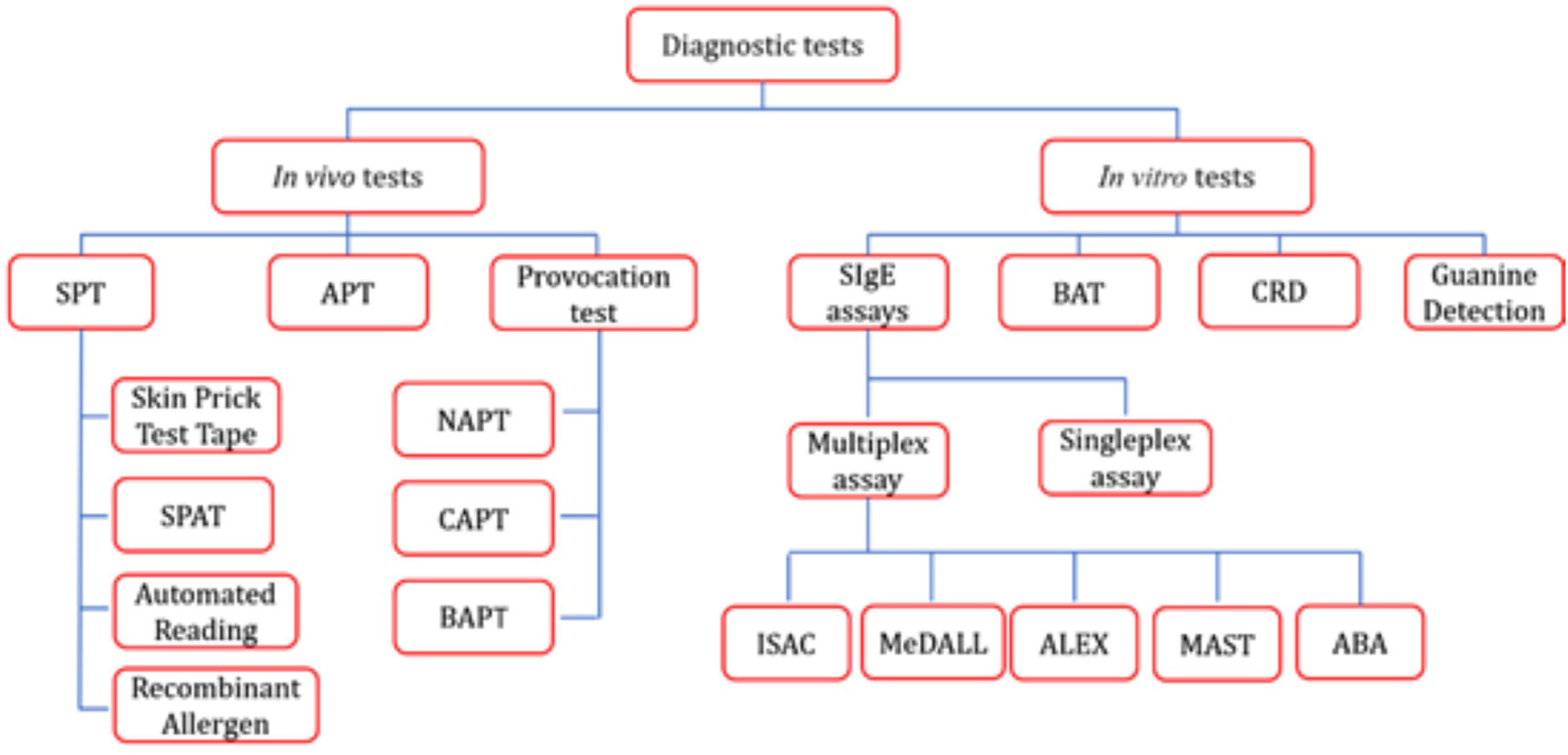
Tree diagram of dust mite allergy diagnostic tests.

In 1964 and 1967, physicians and biologists first elucidated the classification of house dust mites (HDM) and demonstrated that they are the primary source of house dust allergens, thereby revealing the critical role of dust mites in allergic diseases (1). Approximately 1%–2% of the global population—ranging from 65 to 130 million people—is affected (2). HDM unique habits enable them to colonize a wide range of human habitats, and their products predispose them to trigger both innate and adaptive immune responses (3). When dust mite allergens contact the conjunctiva, skin, respiratory tract, or intestinal tract, they can trigger atopic sensitization and related symptoms, including allergic rhinitis, conjunctivitis, asthma, and atopic dermatitis. Dust mite sensitization can be diagnosed based on patient history, SPT, provocation tests, and/or allergen-specific IgE (sIgE) assays, thereby providing a crucial basis for timely treatment and intervention, such as allergen avoidance, pharmacotherapy, and allergen-specific immunotherapy (AIT). This review describes both *in vivo* and *in vitro* detection methods for mite allergy (e.g., Figure 1), analyzes the advantages and disadvantages of each approach, and offers a reference for clinical diagnosis and decision-making as well as for the future development of detection techniques.

## 2 Immunological mechanisms of dust mite allergy

Dust mite allergy can be classified into IgE-mediated and non-IgE-mediated immune responses. In IgE-mediated immune responses, upon exposure to dust mite allergens, the immune system produces sufficient amounts of sIgE antibodies. Non-IgE-mediated immune responses primarily arise from other properties of mite allergens, such as dust mite protein hydrolases (4–10), activators of natural immune response pattern recognition receptors (11), and polysensitization promoters (12, 13).

When environmental dust mite allergens reach a certain concentration, they directly enhance allergen permeability by disrupting tight junctions between epithelial cells and activate epithelial cells to secrete secretion of IL-25, IL-33, and TSLP (thymic stromal lymphopoietin), which activate localized dendritic cells (DCs) and intrinsic lymphocytes (ILC2). DCs recognize dust mite allergens through pattern recognition receptors (e.g., TLR, CLR) recognize dust mite allergens, uptake and presentation of antigens to the lymph nodes. Meanwhile, the protease activity of dust mite allergens inhibits the production of IL-12 by DCs, prompting them to secrete IL-4 and IL-5, which induces the differentiation of Th0 cells towards Th2. IL-33 signaling further strengthens the Th2 polarization ability of DCs. Th2 cells, which are the core of the regulation of allergic responses, secrete key cytokines such as IL-4, IL-5, and IL-13, and their secretion of IL-4 will further reinforce the Th0 cells’ ability to polarize to Th2. IL-4 will further enhance the differentiation of Th0 cells toward Th2 and inhibit the expression of Th1-related genes (e.g., IFN-*γ*) (14, 15). IL-4 drives B-cell IgE class switching by binding to the IL-4 receptor on the surface of the B cell, thereby promoting IgE production (16, 17), and by increasing the expression of CD23 (a low-affinity IgE receptor) on the surface of the B cell, it promotes IgE binding to the B cell and enhances the antigen-presenting capacity, further amplifying the allergic response.

SIgE produced by B cells binds to the surface of mast cells and basophils via the Fc*ε*RI receptor. Upon re-exposure to the allergen, sIgE cross-linking triggers degranulation, releasing mediators such as histamine, leukotrienes (LTs), and prostaglandins (PGD2) and causing acute symptoms (e.g., vasodilatation, smooth muscle contraction). IL-5 promotes the differentiation, survival, and recruitment of eosinophils to the site of inflammation, and eosinophils release major basic protein (MBP), eosinophil cationic protein (ECP), which directly damages the epithelium and secretes IgE. IL-13 and TGF-*β* to promote chronic inflammation and airway remodeling (14). IL-13 induces cuprocyte chemotaxis, increased mucus secretion, and airway hyperresponsiveness. IL-4, IL-5, and IL-13 maintain the activation of Th2 cells and ILC2 through autocrine/paracrine secretion, memory Th2 cells expand rapidly upon re-exposure, and pro-fibrotic factors exacerbate tissue damage and further maintain chronic inflammation.

SIgE production in dust mite allergy requires the breaching of multiple thresholds, including concentration of allergen exposure, Th2 cytokine concentration, and individual immune status. In clinical practice, a sIgE level of 0.35 kUA/L is the threshold for diagnosis of sensitization. SIgE levels below this threshold (e.g., 0.10–0.35 kUA/L) may not trigger clinical symptoms but may indicate a potential sensitization risk. Individual responses are modulated by genetics, environmental microorganisms, and history of previous exposure.

## 3 Detection methods

Dust mite allergen specificity detection methods commonly used in clinical and research settings include two types of tests: *in vivo* and *in vitro*. In *vivo* tests involve the direct application of allergens to the human body, with the reaction observed to make a diagnosis. In contrast, *in vitro* tests involve exposing blood or other bodily fluids to the allergens in isolation and evaluating the reaction based on the results.

### 3.1 *In vivo* tests

#### 3.1.1 SPT

SPT is the simplest *in vivo* tests for assessing IgE sensitization in humans (18). In dust mite sensitization testing, SPT is considered positive if the wheal diameter is at least 3 mm larger than that of the negative control, indicating the presence of mite sIgE in the body (19). Currently, there is no fully standardized criterion for recording and assessment. Most clinical studies measure the wheal size by calculating its mean diameter [(D + d)/2, where D is the largest diameter and d is the diameter perpendicular to D] (20). The criteria for interpreting SPT results are shown in Table 1. The interpretation methods used in Tables 1A, B streamline the evaluation process, enabling rapid screening of allergens and thus are more widely applicable in clinical practice. In contrast, the approaches described in Tables 1C, D provide a quantitative assessment of allergen reactivity, minimize subjective bias, and are therefore more appropriate for scientific research.

**Table 1:** Interpretation criteria for SPT results: A. Ratio Judgment Method Based on Different Criteria; B. Wheal and Erythema Diameter Judgment Method; C. Skin Index (SI, SI = Average diameter of allergen wheals/Average diameter of wheals in the positive control group) Judgment Method; D. Other Judgment Criteria.

A.	
Ratio range	Interpretation
0%–25% or equal to negative control	(-)
26%–50%	(+)
51%–100%	(++)
101%–200%	(+++)
Greater than 200%	(++++)

B.	
Wheal diameter (S)	Interpretation
Greater than 3 mm (compared to negative control)	Positive
5 mm–9 mm	(+)
10 mm–14 mm	(++)
15 mm–19 mm	(+++)
Greater than 20 mm	(++++)

C.	
Skin index (SI) range	Interpretation
< 0.5	(-)
0.5 ≤ SI < 1.0	(++)
1.0 ≤ SI < 2.0	(+++)
≥2.0	(++++)

D.	
Criteria	Interpretation
No reaction or equal to negative control group	(-)
Ratio of histamine wheal ≥ 1/4 of the area	(+)
≥1/2 of the positive control area	(++)
Equal to positive control	(+++)
Greater than 2 times the positive control area	(++++)

When positive SPT results are combined with patient history, IgE-mediated allergic diseases can be definitively diagnosed with a positive predictive value of 95%–100% (21–23). This method offers advantages such as ease of operation, rapid visualization of results, time efficiency, reproducibility, cost-effectiveness, and high sensitivity. SPT is generally safe, with few adverse reactions—typically localized to the skin and very rarely systemic (24, 25).

The disadvantages of SPT primarily stem from a high rate of false-positive and false-negative results, which are attributed to factors such as the operator's skill, the type and shape of the puncture device, and the applied force (26). SPT results are also influenced by medications such as antihistamines, tricyclic antidepressants, tranquilizers, anti-IgE monoclonal antibodies, and H2 receptor antagonists. The measurement of wheal size involves a certain degree of subjectivity, and there are time differences in measurement and assessment among subjects of different ages and body mass indices (27). The lack of standardization in selecting antigen reagents and determining puncture reagent concentrations can also affect results. SPT can cause anxiety and pain in some subjects, particularly younger children (28).

Recent developments in SPT for clinical applications and research include innovations such as skin prick tape, which is less painful and more acceptable while reducing cross-contamination during multi-allergen testing and offering similar biological effects; however, it has not yet been fully empirically validated for clinical use (29). The Skin Prick Automated Test (SPAT) device (30) demonstrates higher reproducibility and tolerability, reduces human error, and decreases experimental variability compared to conventional SPT. It also saves testing time and reduces the consumption of allergen solutions (31). Technologies such as 2D scanners, blood flow measurements, skin impedance, thermography, photography, and 3D scanners enable automated reading of test results; however, issues related to time, cost, and accuracy currently limit their use in clinical practice (32). The use of pure allergens overcomes batch-to-batch variability in natural allergen extracts by offering a clear composition, high purity, and the removal of non-allergenic components, thereby improving test specificity and reducing cross-reactivity (33).

#### 3.1.2 APT

APT uses protein allergens known to elicit an IgE-mediated immediate-type allergic reaction and evaluates the test site for an eczematous delayed-type reaction after 48–72 h (34, 35). It can be used for allergen detection in hay fever, asthma, urticaria, atopic dermatitis, etc. APT has a high degree of specificity and is an important tool for identifying allergens that cause atopic eczema and dermatitis syndrome (AEDS) (36), and it is also suitable for identifying atopic dermatitis caused by mite allergy (37). A study found that patients with both endogenous and exogenous atopic dermatitis (AD) showed a positive response to APT to house dust mites (38). APT has been used in the detection of mite allergens associated with respiratory diseases mainly to assess its value in the diagnosis of allergic rhinitis and asthma. APT is able to recapitulate the pathophysiology of the T-cell-mediated allergic response, and in children with allergic rhinitis or asthma patients showing high positivity rates ranging from 25% to 56% (39). In dust mite allergy testing, approximately 10% of patients are positive only by APT, avoiding misclassification as non-allergic if negative in conventional SPT or *in vitro* IgE testing and reducing the risk of untimely intervention or inappropriate management. APT has a high safety profile, with fewer side effects, most of which are mild reactions such as localized skin rashes, contact urticaria, and localized pruritus (40).

APT also has limitations in mite allergy testing. Standardizing the substances, concentrations, vehicles, interpretation times, and procedural techniques used in the APT is difficulty (41); skin conditions at the test site and age differences also affect APT results, adult and adolescent patients reacting positively to APT for mite allergens significantly is more often than children (38). Pharmacologic factors such as steroids, cyclosporine A, tacrolimus, and antihistamines can affect the test results; the test itself is time-consuming; and the stimulus reaction of the APT itself may also lead to false-positive results. And heterogeneity between different studies, although APT shows higher sensitivity and specificity in some cases, test results should be interpreted with caution (37).

#### 3.1.3 Allergen provocation test

Allergen provocation test is one of the most important methods for diagnosing allergic diseases, which can visually demonstrate the clinical correlation between allergens and the symptoms and severity of allergic diseases. When the history suggests allergy and serum sIgE is not detected or SPT is negative, provocation tests are feasible (42–44). The provocation tests used for mite allergy detection include nasal allergen provocation test (NAPT), conjunctival allergen provocation test (CAPT), and bronchial allergen provocation test (BAPT).

The NAPT is currently the only available test to confirm nasal reactivity to allergens. It is safe and highly reproducible (45). NAPT is a valuable test for confirming the diagnosis of dust mite allergy when the SPT test result is negative, and the symptoms following NAPT for dust mites are also of high value in predicting perennial allergic rhinitis (46). Compared to SPT, dust mite NAPT has a lower sensitivity and higher specificity in the diagnosis of allergic asthma (47).

The CAPT is the only test capable of determining the relationship between ocular manifestations and sIgE, with a diagnostic sensitivity and specificity of 90% and 100%, respectively, in a study to diagnose HDM-induced allergic conjunctivitis, attesting to its high antigenic quality (48). The CAPT can provide valuable clinical information, and the lack of more thorough evaluation of safety aspects has not been fully utilized in practice (49). CAPT can provide valuable clinical information, lacking a more thorough evaluation of safety, and is not fully utilized in practice (46).

Dust mite allergen is one of the common allergens in many patients suffering from asthma and co-morbid AR (50), and BAPT is one of the most important tools for the diagnosis of allergic asthma (51–53). The absence of standardized protocols and equipment in bronchial provocation testing diminishes its reproducibility. Moreover, bronchial provocation testing exhibits lower safety compared to other *in vivo* tests, as it may trigger adverse effects—including acute bronchospasm, asthma attacks, laryngeal edema, and, in rare cases, anaphylactic shock—which further restrict its clinical use (54). A study proposed the use of NAPT instead of BAPT as a diagnostic tool by comparing bronchial and nasal allergen provocation tests in patients with bronchial asthma and mite sensitization, and showed that NAPT could be used to confirm the relevance of HDM sensitization in the majority of asthma cases prior to BAPT; in NAPT-negative patients, the use of BAPT was still recommended to rule out an HDM-induced asthma reactions (55).

The provocation tests have high sensitivity, specificity and validity, and accuracy is also high relative to skin tests. Because of its time-consuming operation, high cost and equipment requirements, technical difficulty, and the need for skilled personnel for operation and measurement, provocation tests are generally not used as an initial screening tool for allergy, instead has been more widely used in the study of pathogenesis and pathophysiology (56, 57), and is also used to assess the effectiveness of treatments such as the efficacy assessment of immunotherapy for house dust mite allergens (58).

### 3.2 *In vitro* test

#### 3.2.1 BAT

Basophils and mast cells are the key effector cells of immediate allergic reaction. The process of basophil degranulation is known as basophil activation. With the development and popularization of flow cytometry, and the discovery of unique markers such as CD63, CD203, and unique markers for identifying basophils, the BAT has gradually become a universally accepted auxiliary allergic reaction detection method (59). The BAT measures the expression of activation markers on the surface of basophils by means of flow cytometry, for example, CD63, a membrane protein localized to the same secreted lysosomal granules containing histamine, it is a precise marker for allergenic desmoplasia by regulating cytokinesis after allergen-mediated activation of mast cells and basophils (60), and the release of histamine in the activation of basophils correlates well with the upregulation of CD63, which was measured by flow cytometry on the CD63 expressed on basophils is detected and evaluated by flow cytometry to determine whether basophils are activated and the level of activation to make a diagnosis of allergy. Common basophil recognition markers and activation markers are shown in Table 2 (61).

**Table 2:** Basophils recognition and activation markers.

Marker	Description and gating strategy
Identification markers	
CCR3	Stable marker used for identification.
CD203c	Widely used identification marker; represents degranulation of basophils.
CD123	Highly expressed on basophils.
IgE	—
CRTH2	Differentiates eosinophils via lateral scatter.
Activation markers	
CD63	Widely used activation marker and an accurate indicator of allergic degranulation.
CD107a, CD107b	Expressed only by activated basophils; their upregulation is similar to CD63.
CD69	—
CD13	—
CD164	—
p38 MAPK, STAT5	Intracellular phosphorylation markers used to measure basophil activation.

The utility of basophil activation test in dust mite allergy has been studied and analyzed through natural extracts, purified extracts, and recombinant allergen fractions of dust mites, and its overall performance is good (62). This test reduces the risk of severe allergic reactions by detecting 150–2,000 basophils in less than 0.1 ml of fresh blood in response to allergen crosslinked IgE, which is more reproducible and less stressful for the patient compared to other tests.

The limitations of BAT are: ideally, whole blood BAT should be performed within 4 h after blood collection to maximize basophil viability and function, because basophil reactivity decreases significantly with time, when it appears that it takes a long time from blood collection to BAT, blood needs to be processed and preserved, and there is no standardized time and conditions for preservation, and the optimal preservation conditions need to be further explored and researched; whole blood BAT can be interfered by serum components such as blocking antibodies; basophil enrichment and purification can cause cell loss and *in vitro* activation, which can affect the results of the assay; the source of allergens is another key factor in the application of BAT in clinical and research applications, and there is also the problem of the lack of standardization of allergens (61); systemic application of steroids and cyclosporine A can affect the results of the BAT assay as well (63); how to choose the gating strategy for identifying basophils according to different conditions also needs to be further investigated (64); additionally, the high cost, specialized equipment, and requirement for trained personnel limit the clinical application of the basophil activation test (BAT). The clinical application of BAT still needs to be further optimized and standardized, especially in the control of allergen selection and pharmacological interventions; and cost barriers can be mitigated by sharing equipment, optimizing processes, and implementing standardization.

#### 3.2.2 Sige test

The serum sIgE test detects IgE antibodies against specific allergens (e.g., dust mites) in patients' serum using *in vitro* immunological techniques. Studies have demonstrated that the sIgE test for dust mite allergy has a sensitivity of 85%–98.8% and a specificity of 89.6%–97.9%, with a significant positive correlation with the skin prick test (SPT) (*r* = 0.506–0.737) (65, 66). Additionally, the sIgE test can be quantitatively graded: an sIgE level of ≥0.35 kUA/L is considered positive, while a level of ≥3.5 kUA/L (grade 3) indicates moderate-to-severe sensitization, which partially correlates with clinical symptom severity (67). Compared with other detection methods, the six-class classification of sIgE provides an objective standard for allergy diagnosis by quantifying the degree of sensitization, and has become a key tool for AIT. Additionally, sIgE testing eliminates the confounding effects of skin condition, age, and medication use on test results, and it is associated with a very high safety profile (68).

Currently, more than 4,000 scientific articles have demonstrated the clinical value of ImmunoCAP, which is considered the “reference standard” for *in vitro* IgE detection (69). With the emergence and development of allergenic molecules, the application of this test has introduced allergen research into the field of precision medicine (70). The ImmunoCAP test for individual allergens is based on the coupling of sIgE from serum or other body fluids to solid-phase allergens, followed by detection of bound sIgE using enzyme-labeled anti-human IgE, with the level of sIgE indicated by fluorescence intensity.

The main advantages of the ImmunoCAP assay for individual allergens are the quantitative detection of allergen-specific antibodies based on the total IgE standard calibration system of the WHO human reference preparation; by immobilizing a larger number of allergens on the surface of the ImmunoCAP to ensure complete binding of the antibodies, the high sensitivity of the assay and a wide linear detection range are achieved, with good precision and reproducibility. The limit of detection is as low as 0.1 kUA/L (range 0.1–90 kUA/L); there is no interference from allergen-specific IgG antibodies, which improves the accuracy of the IgE assay and somewhat reduces the use of provocation tests, etc., in the diagnosis of allergies; a retrospective study has shown that the ImmunoCAP testing is the most suitable standalone method for confirming allergies to nuts, wheat, and other specific foods. Additionally, it is applicable for detecting allergic reactions to a broad range of allergens (71); its limitations are mainly the small number of allergen molecules available, the incomplete spectrum of IgE responses obtained from a single or a few tests, and the high cost of multiple tests and the large amount of serum samples required.

The proof-of-concept that proteomics microarray methods can be applied to the diagnosis of allergic sensitization was validated in 2002 (72), and subsequent literature has successively validated the same arrays (73–75), commonly referred to as the Immuno Solid-phase Allergen Chip (ISAC) system, which is based on the same principles as the individual ImmunoCAP assay test. The ISAC assay is a highly reproducible and accurate method (76), as a more complete assay platform, ISAC can simultaneously measure sIgE against more than 100 allergens with a micro-volume of serum, while its assay performance is stable and has been evaluated at 23 sites worldwide by different operators, essentially obtaining the same results irrespective of the analytical site, laboratory conditions, operator and microarray batch, and to a certain extent, can distinguish cross-reactivity. The disadvantages of the ISAC assay include lower analytical sensitivity and higher cost per assay, which limits its use in allergy research. Due to the high cost of the test, ISAC testing is currently only performed in a subset of the population in most clinical services in the UK, i.e., patients whose diagnosis remains unclear after SPT and ImmunoCAP testing. However, some researchers still believe that the test is expected to become routine (77).

Microarray technologies have been progressively refined, incorporating recombinant allergens (78), and leading to the development of platforms such as the MeDALL chip, Allergy Explorer (ALEX), Multiple Allergen Simultaneous Testing (MAST), Allergen Micro-Bead Array (ABA), and a novel immunofluorescence chromatography strategy (D-FILA). The MeDALL chip has demonstrated higher sensitivity in detecting sensitizations compared to ImmunoCAP sIgE or SPT (79, 80). ALEX employs nanoparticle technology to immobilize a comprehensive panel of allergen extracts and molecular components on a solid phase, enabling both second-level diagnostics (represented by extract allergens) and third-level diagnostics (represented by single molecules) (81), and is associated with the Allergenius system developed for the interpretation of ISAC results, making it a good diagnostic tool for “bottom-up” allergy diagnosis (82). MAST based on immunoblotting techniques, such as EUROLINE, also represents a valid diagnostic option since MAST and ImmunoCAP were found to be in general agreement with respect to inhalant, food and venom allergens when compared to ImmunoCAP (83). ABA are a good diagnostic tool in the diagnosis of allergies (84), which quantifies IgE binding levels by flow cytometry detection of fluorescent signals from microbeads, ABA can be used to detect IgE responses to inhalant allergens such as dust mites (e.g., Der p 1, Der s 1) and pollen (e.g., Bet v 1, Phl *p* 5), which can help in the diagnosis of allergic rhinitis and asthma (84, 85). D-FILA based on quantum dot immunochromatography has also been used for sIgE detection, especially for detection of dust mite allergy has a higher sensitivity and accuracy compared to the conventional ImmunoCAP detection system and does not require stringent patient conditions and specialized equipment, which reduces the economic burden for both laboratories and patients (86).

#### 3.2.3 CRD

CRD is a specific diagnostic method based on recombinant or purified allergen components, which is centered on the precise identification of sensitizing proteins through the detection of sIgE against a single allergen component (rather than crude extracts) in the patient's serum (87). Compared to other diagnostic methods, specific sensitizing components can be precisely identified. There are significant differences in the sensitizing proteins of dust mites in different regions and populations, and CRD of dust mite allergens makes up for the lack of other diagnostic strategies in accurately identifying the sensitizing fractions, which is an important guide to accurately formulate dust mite immunotherapy protocols. The recombinant or purified dust mite allergen fractions that are currently widely used in clinical or research applications are shown in Table 3. The current HMD CRD mainly cover the core fractions Der p 1, Der p 2, and Der p 23, which have been validated for their high specificity and phenotypic correlation, and the minor fractions Der p 5, Der p 7, and Der p 21, which have complementary value in specific populations (e.g., patients with negative conventional tests). In the future, the clinical significance of emerging components (e.g., Der p 11, 18) needs to be further validated and the standardization of multi-component combination assays needs to be promoted.

**Table 3:** Recombinant or purified dust mite allergen components in clinical or research applications.

Allergenic component	Species origin)	Biological function	Clinical significance	Value of application in CRD
Der p 1	D. pteronyssinus	Cysteine protease, protease activity and epithelial barrier disruption (90), activation of inflammatory signaling pathways (91, 92),immunomodulatory effects (92–94)	HDM core allergen (94), positivity rate 70–90%, concentration positively correlated with asthma severity (95, 96)	Distinguish cross-reactivity, accurate typing (97, 98)
Der f 1	D. farinae	Cysteine proteases, activation of Th2-type immune responses, adjuvant effects (94, 99)	Dust mite core allergens for differentiating HDM from dust mite sensitization	Specific detection of dust mite allergy to guide immunotherapy target selection (100–102)
Der p 2	D. pteronyssinus	NPC2 family proteins, activation of the TLR4 pathway, lipid binding and molecular mimicry, regulation of Th2 immune preference (103–106)	Core sensitizer, synergistic sensitization with Der p 1, >80% positive (107, 108), cross-reactivity (98)	Combined Der p 1 improves diagnostic sensitivity and predicts allergic phenotypes (109, 110)
Der f 2	D. farinae	NPC2 family proteins, immune activation and polarization of Th2-type immune responses (92, 103, 104)	Dust mite sensitization markers that correlate with asthma severity (111)	Distinguishing mite species-specific sensitization and optimizing desensitization regimens
Der p 23	D. pteronyssinus	Epidermal proteins, binding mite fecal pellets, association with innate immunity (112)	Novel highly sensitizing component, 60–70% positivity, significant correlation with asthma severity (113, 114), associated with severe rhinitis	Complementing the omissions of traditional tests to improve diagnostic coverage (113, 115); differentiating primary sensitization from cross-reactivity (115)
Der f 24	D. farinae	Belongs to the Alpha-Actinin family of proteins, has a molecular weight of 90 kDa, and mediates the IgE allergic response (116)	Newly identified sensitizing components strongly associated with allergic asthma and allergic rhinitis (117)	Exploratory diagnostic markers; for investigational CRD testing; improving diagnostic coverage
Der p 10	D. pteronyssinus	Promyosin (heat-stable protein); cross-reactivity and immune activation (118)	Cross-reactivity with crustaceans (shrimp, crabs) (119)	Identifying Multiple Sensitization Risks to Avoid Misdiagnosis of Simple Dust Mite allergy
Der p 5	D. pteronyssinus	Dimeric structure, non-protease-dependent pathway of immunostimulation activation, lipid carriers (68, 120, 121)	Closely related to asthma (122)	Helps distinguish cross-reactions (122)
Der p 7	D. pteronyssinus	Lipid transport protein, function unknown (123)	Secondary allergens, associated with asthma and allergic rhinitis	
Der p 21	D. pteronyssinus	Activation of Toll-like receptor 2 (TLR2) triggers innate immune response (124)	Secondary allergens, with higher positivity rates among moderately severe patients (111)	
Der p 11	D. pteronyssinus	paramyosin	Related to Atopic Dermatitis (125)	
Der p 18	D. pteronyssinus	Chitinase activity (126)	minor allergen	

CRD employs molecular-level identification of key house dust mite allergen components to resolve the cross-reactivity and component ambiguity inherent in traditional extract-based assays. Its core values include: precise differentiation of cross-reactive IgE responses to minimize misdiagnosis; prediction of disease severity and risk of complications; and guidance for personalized AIT to achieve significantly enhanced treatment efficacy. Patients selected for AIT based on CRD profiles demonstrate significantly higher response rates compared to those chosen via conventional diagnostic methods (88).

#### 3.2.4 Environmental allergen testing—guanine testing

Guanine is the end product of nitrogen metabolism in dust mite feces, and its content is significantly and positively correlated with dust mite population density and the concentration of key allergens (e.g., Der p 1). The guanine test is suitable for the rapid assessment of dust mite contamination in households and public places. It is easy to perform, requires no specialized equipment and is suitable for use by non-technical personnel. The results are presented in a semi-quantitative form to facilitate risk classification, detection of dust mite allergens (e.g., Der p 1) or guanine in household dust, and assessment of exposure risk. The guanine test is low-cost and low-cost and time-efficient (results in 15–20 min). However, guanine is also a metabolic byproduct of various arthropods, and biological residues containing guanine can also be found in the environment; these factors may all affect the accuracy of results, resulting in false positives, and it has low sensitivity (limit of detection about 500 ng/g dust) (89).

## 4 Conclusion and prospects

Technological advances in testing have rendered detection methods more convenient, accurate, and efficient. At the same time, there are many bottlenecks and challenges. The extraction of allergens used for testing, molecular components, operation, detection thresholds and units of different testing methods have not yet been fully standardized, which affects the comparability of results; dust mite allergens contain more than 40 protein fractions, which cannot be fully covered by traditional testing methods, and at the same time, there is cross-reactivity, which makes accurate identification difficult; the development of new technologies is rapid, and there is a lack of clinical validation; the existing tests cannot reflect the change of allergic status or treatment effect in real time; in the future, it is necessary to break through the bottlenecks of cross-reactivity, technology standardization and resource accessibility, high cost, and to optimize the whole chain from diagnosis to management by relying on multi-omics, nano-materials and artificial intelligence.

Although the diagnostic methods for dust mite allergy discussed in this review are supported by existing literature, some studies may exhibit methodological biases due to limitations in sample size, geographic variability, and testing standards. Furthermore, discrepancies in result interpretation and evaluation criteria across different studies underscore the need for future research to establish standardized diagnostic protocols and assessment systems.

Overall, *in vivo* and *in vitro* tests have their own advantages and disadvantages. With technological advances and deeper interdisciplinary cooperation, future testing platforms are expected to achieve multimodal data fusion, which is truly accurate, intelligent, and individualized, providing more comprehensive and reliable support for early diagnosis, treatment monitoring, and prognosis assessment of dust mite allergy.
